# Programmed frozen embryo transfer cycles are associated with a higher risk of abnormal placental development: a retrospective cohort study of singleton live births

**DOI:** 10.3389/fendo.2023.1202044

**Published:** 2023-06-06

**Authors:** Fuxin Wang, Qi Wang, Ye Song, Jie Ding, Hong Li, Qingxia Meng

**Affiliations:** ^1^ Center of Human Reproduction and Genetics, The Affiliated Suzhou Hospital of Nanjing Medical University, Suzhou, China; ^2^ Suzhou Municipal Hospital, Gusu School, Nanjing Medical University, Suzhou, China; ^3^ Department of Obstetrics, The Affiliated Suzhou Hospital of Nanjing Medical University, Suzhou, China

**Keywords:** frozen-embryo transfer, endometrial preparation regimen, hormone replacement treatment, natural cycle treatment, placental disorder

## Abstract

**Introduction:**

Abnormal placental development can lead to adverse outcomes for both mother and fetus. The effect of different types of endometrium preparation regimens of frozen-thawed cycles on the placental development features associated with the perinatal outcomes remains unclear. Hence, we conducted a retrospective cohort study to assess the impact of specific endometrial preparation regimens on placenta-mediated pregnancy complications in singleton live births.

**Methods:**

A retrospective cohort study was conducted evaluating data of all singleton live births both conceived naturally or by *in vitro* fertilization (IVF) therapy from 2018 to 2020 at our hospital. Two exposed groups of frozen-thawed embryo transfer (FET) were created by the endometrium preparation regimen as the modified natural cycles (mNC) and the programmed cycles. The nonexposed group was the singleton pregnancies conceived naturally. The obstetrical and perinatal outcomes were compared among the three groups using multivariate analysis to adjust the results for determinants potentially associated with the abnormal placental development.

**Results:**

A total of 2186 pregnant women with singleton live births were included in our final analysis and were divided into three groups as naturally conceived group (n=1334), mNC-FETs group (n=217) and programmed-FETs group(n=635). After adjusting for maternal age and parity, no significant difference was observed on the risk of placental disorders between mNC-FET cycles and natural conceived pregnancies (aOR 1.16; 95%CI 1.31-7.01), while programmed-FET cycles were associated with a higher occurrence of placental disorders (aOR 5.36; 95%CI 3.63-8.05). Using the mNC-FET group as a reference and adjusting for confounders such as maternal age, parity, endometrial thickness, and number of embryos transferred, we found that the main manifestation of abnormal placentation in programmed FET cycles was abnormal placental attachment, including placental adhesion and placenta increta (aOR 2.50, 95%CI 1.36-4.90). The dysfunction of placentation in programmed-FET cycles was independently associated with the type of infertility, the total dose of Femostone and thinner endometrium. Additionally, placental disorders in the programmed-FET group were associated with higher rate of preeclampsia, postpartum hemorrhage and Cesarean section.

**Conclusion:**

Our retrospective study revealed that the programmed-FET has a substantial impact on placental development, resulting in a higher incidence of preeclampsia, postpartum hemorrhage and Cesarean section. These findings have significant implications on clinical decision-making.

## Introduction

With the improvements in cryo-techniques, the cycles of frozen embryo transfers (FETs) have increased dramatically since 1983. FETs are beneficial not only for reducing the rate of ovarian hyperstimulation syndrome (OHSS), but also for allowing time for preimplantation genetics testing and facilitating fertility preservation (1).

Although the pregnancy rates after cryothawing and fresh embryo transfer are similar, the effect of embryo cryopreservation on obstetric and neonatal outcomes is still under debate. It has been reported that compared to fresh embryo transfer, FET has been shown to result in lower rates of low birth weight and premature birth, but higher rates of hypertensive disorders during pregnancy, induced labor, cesarean section, and lower Apgar scores of the newborns (2–4).

Despite the cyro-technique itself, the different endometrial preparation protocols used for FETs in daily practice are crucial to the outcome of newborns. There are three commonly used endometrial preparation regimens for FETs, natural cycles, modified natural cycles (mNC) and programmed cycles. The different endometrial preparation programs will result in different endometrial environments and changes in implantation and placental formation (5). Placental data add to the understanding of intrauterine processes and a comprehensive assessment of placental histopathological patterns may shed light on the potential causes of different pregnancy outcomes in different endometrial preparation regimens of FET pregnancies. Recently, it has been reported that embryo vitrification has a significant effect on the placental histopathology pattern and is associated with a higher prevalence of dysfunctional labor (6). Meanwhile, there is a higher rate of anatomic and vascular placental pathology of pregnancies arising from frozen embryo transfers than those from fresh transfers (7). However, only a few studies have evaluated the impact of different endometrial preparation protocols of FET on placental development and perinatal outcomes (6, 8, 9).

The aim of our study is to assess the effects of different endometrial preparation regimens of FETs on the incidence of placental disorders and the perinatal outcomes of single birth.

## Materials and methods

### Study design and patients

This was a retrospective study that evaluated data of all singleton live births, both conceived naturally or through *in vitro* fertilization (IVF) therapy, at Suzhou Municipal Hospital and the Center for Reproduction and Genetics at Suzhou Municipal Hospital, Jiangsu Province, from January 2018 to December 2020. The study protocol was approved by the institutional review board of the hospital. Data were collected from medical records, including baseline characteristics, treatment-related information and reproductive outcomes reported up to live birth. Multiple pregnancy and fresh cycles of IVF were excluded.

### Endometrium preparation before embryo transfer

In our center, there are mainly two endometrial preparation programs based on patient preference or the discretion of physician, the mNC-FET and programmed-FET.

mNC-FET refers to a modified natural cycle, as a dose of human chorionic gonadotropin (c) trigger is given based on ultrasonic measurements of the dominant follicle and luteal support was also administered. The day of ovulation was confirmed by transvaginal ultrasound. Luteal phase support started on the day after ovulation with oral dydrogesterone (Duphaston; Abbott, OLST, Netherlands) at a dose of 30mg three times daily. Cleavage-stage embryo or blastocyst-stage embryo were thawed and transferred on day 3 or 5 after ovulation.

For patients in programmed-FET group, oral estradiol valerate (Progynova; Bayer Schering Pharma AG, Germany; at a dose of 2-3mg twice a day) was given on the second or third day of menstrual cycle, after confirming that patients were in the early proliferative phase for menstrual cycle. When the endometrial thickness reached at least 7 mm, vaginal progesterone gel at a dose of 90 mg once daily or micronized progesterone at a dose of 400 mg twice daily combined with oral dydrogesterone at a dose of 30 mg three times daily was added. If the thickness of the endometrium could not meet 7 mm, the dose of oral estradiol valerate was increased to 8 mg or by adding of one or two vaginal 17-βestradiol tablets (Femoston; Duphaston; Abbott, OLST, Netherlands, brick red tablets containing 2mg estradiol). FET was scheduled for 4 days for cleavage-stage embryos and 6 days for blastocyst-stage embryos from the starting of progesterone.

### Placental examination

**Figure 1:**
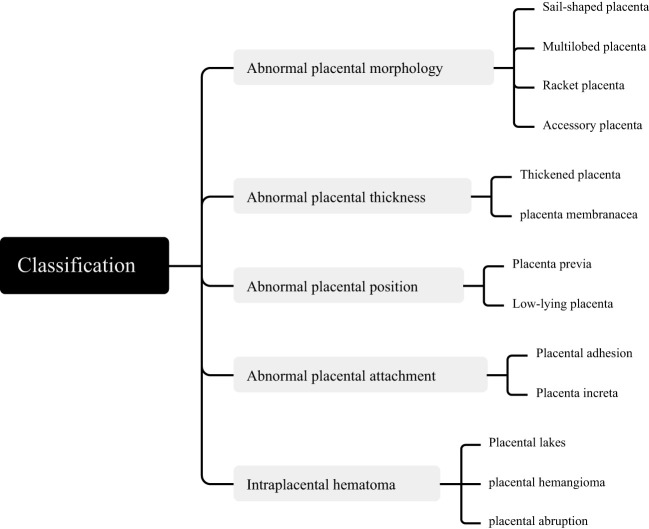
Classification of placental developmental abnormalities.

Macroscopic placental examinations were performed for all deliveries in accordance with the institutional protocol employed during the study period. The evaluation of placental morphology and structure included assessment of placental size and weight, umbilical cord, fetal and maternal surfaces, and placental parenchyma. Any abnormalities detected were documented. The main placental disorders found in our study are shown in **Figure 1**.

### Study outcomes

We obtained information on obstetric complications from the medical records and defined diagnoses according to the International Classification of Diseases and Related Health Problems (ICD), 10^th^ revision (ICD-10). Hypertensive disorders in pregnancy were defined as the O13–15 code (pregnancy-induced hypertension, preeclampsia, and eclampsia); preeclampsia, O14; PPROM, O420; placenta previa, O44; placental abruption, O45; induction of labor, O61; postpartum hemorrhage (PPH), O72 with bleeding > 500 mL; and CS, O82. Preeclampsia was defined as gestational hypertension combined with proteinuria and/or organ dysfunction. Intrahepatic cholestasis of pregnancy was defined as O26. Gestational diabetes was defined as O24.9. All diagnoses were allocated by medical doctors. Perinatal outcomes evaluated were child’s sex, gestational age (post-term birth [> 42 weeks], preterm birth [PTB; <37 weeks]), birth weight (low birthweight <2500g, high birthweight>4000g)), small gestational age (SGA), and large gestational age (LGA), which were defined as <–2 standard deviation and >+2 standard deviation difference from the expected sex-specific birth weight for the given gestational age, respectively. In all groups gestational age was calculated based on a first-trimester ultrasonography scan.

### Statistical analysis

Our main comparison was programmed-FET versus mNC-FET. In addition, we also compared the programmed-FET group, mNC-FET group to the natural conceiving group. All statistical analyses were performed with IBM SPSS Statistics (version 25.0) or the free software computing environment R (version 4.1.0). Data distributions were evaluated with the Shapiro-Wilk test. Normally distributed data were expressed as mean ± standard deviation (SD) and non-normally distributed data were expressed as median (interquartile range). A nonparametric test (Kruskal-Wallis test) was used to compare the rank means between multiple groups of skewed distributions. After the Kruskal-Wallis test, if the differences were statistically significant, the rank sum test for multiple comparisons was continued to be completed. Categorical information was expressed as the number of cases (as a percentage of the total) and assessed by Pearson’s chi-square test or Fisher’s exact probability method. Univariate logistic regression analysis was used to determine the various factors affecting placental development and multivariate logistic regression analysis was used to adjust for confounding factors to investigate the effect of relevant factors on abnormal placental development during hormone replacement cycles. P-value < 0.05 was considered statistically significant.

## Results

### Baseline characteristics

**Figure 2:**
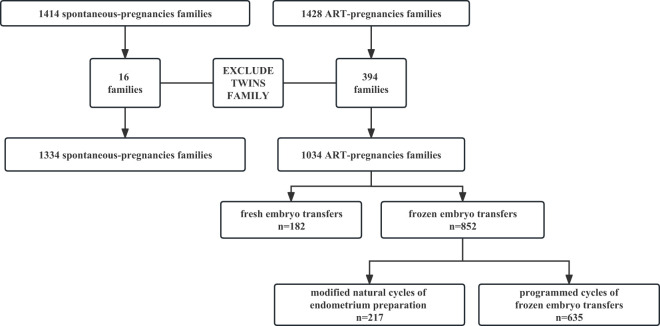
Flowchart of patients’ selection process.

A total of 2,186 patients who met the inclusion and exclusion criteria were included in this analysis. Of these, 1334 were conceived naturally, 217 were conceived through mNC-FET cycles and 635 were through programmed-FET cycles, respectively (**Figure 2**). The baseline characteristics were shown in **Table 1**. The maternal and paternal ages were differed among the 3 groups, with the youngest in the naturally conceived group and oldest in the modified natural cycle group.

**Table 1:** Demographic, clinical and IVF treatment characteristics.

Variables	Naturally conceived	Frozen embryo transfers		
		Modified natural cycle	Programmed cycle	*P-value*
	(n=1334)	(n=217)	(n=635)	
Maternal age (years)	28.9 (4.8)	31.1 (5.6)	30.2 (5.1)	<0.001***
Paternal age (years)	30.2 (5.4)	32.2 (6.9) *	31.4 (6.0) *	<0.001
Maternal BMI (kg/m^2^)	20.8 (3.1)	21.5 (3.9) *	21.8 (4.1) *	<0.001
Parity (n (%))				<0.001***
• first	983 (73.7)	181 (83.4)	586 (92.3)	
• high order	351 (26.3)	36 (16.6)	49 (7.7)	
Duration of infertility (m)		33.0 (27.0)	34.0 (27.0)	<0.001
Type of infertility				0.144
• primary infertility		112 (51.6)	364 (57.3)	
• secondary infertility		105 (48.4)	271 (42.7)	
Cause of infertility				0.103
• female		105	320	
• male		28	83	
• mixes		74	209	
• unexplained		9	66	
b-AMH (ng/mL)		3.6 (3.2)	4.9 (5.3)	0.859
Total antral follicle count		14 (8.75)	15 (8)	0.071
EMT (mm)		9.7 (2.6)	9.0 (2.0)	0.002
Embryo stage at transfer				0.863
• Cleavage stage		68 (31.3)	195 (30.7)	
• blastocyst		149 (68.7)	440 (69.3)	
No. of embryos transferred				<0.001
• single		135 (62.2)	242 (38.1)	
• double		79 (36.4)	383 (60.3)	
• triple		3 (1.4)	10 (1.6)	

BMI, body mass index.

AMH, anti-Müllerian hormone.

E_2_, serum estradiol.

EMT, endometrial thickness.

Skewed data were presented as median (with interquartile range).

Categorical data was expressed as the number of cases (as a percentage of the total).

P-value <0.05 was considered statistically significant.

*Significantly different from the naturally conceived group.

**Significantly different from the naturally conceived group and natural cycle group.

***Significant differences were found between any two groups.

### Maternal and neonatal outcomes

The maternal and neonatal outcomes were categorized by the type of conception and the type of endometrial preparation protocols (**Table 2**). There were no statistically significant differences in the rates of intrahepatic cholestasis of pregnancy, fetal intrauterine growth restriction, and the Apgar score among the three groups. Compared to naturally conceiving group, the rates of gestational diabetes, preeclampsia, Cesarean section, premature birth and postpartum hemorrhage were significantly higher in the FET groups. Furthermore, the incidences of preeclampsia and postpartum hemorrhage were highest in the programmed FET group with statistically significance. The rates of low birth weight and high birth weight were both increased in the programmed-FET group.

**Table 2:** Obstetric and perinatal outcomes.

Variables	Naturally conceived	Frozen embryo transfers		
		Modified natural cycle	Programmedcycle	*P-value*
	(n=1334)	(n=217)	(n=635)	
Maternal complications during pregnancy (n (%))				
• Gestational diabetes	307 (23.0)	69 (31.7)*	180 (28.3)*	0.003
• Preeclampsia	23 (1.7)	5 (2.3)*	39 (6.1)**	<0.001
• Intrahepatic cholestasis of pregnancy	9 (0.7)	4 (1.8)	9 (1.4)	0.146
• Fetal intrauterine growth restriction	9 (0.7)	0 (0.0)	5 (0.8)	0.222
Gestational week of delivery (weeks)	39 (1.3)	39 (2.0)*	39 (2.0)*	<0.001
Delivery mode (n (%))				<0.001***
• Vaginal delivery	842 (63.1)	92 (42.4)	186 (29.3)	
• Cesarean section	492 (36.9)	125 (57.6)	449 (70.7)	
Premature birth rate (n (%))	43 (3.2)	13 (6.0)*	47 (7.4)*	<0.001
Postpartum hemorrhage (n (%))	89 (6.7)	10 (4.6)	95 (15.0)	<0.001***
Neonatal outcomes				
• Apgar1' (mean score)	10 (0)	10 (0)	10 (0)	0.968
• Apgar5' (mean score)	10 (0)	10 (0)	10 (0)	0.978
• Birth weight (g)	3360 (520)	3350 (600)	3450 (585)*	<0.001
• Low birth weight (n (%))	28 (2.1)	8 (3.7)	25 (3.9)*	0.048
• high birth weight (n (%))	81 (6.1)	14 (6.4)	63 (9.9)*	0.008

Skewed data were presented as median (with interquartile range).

Categorical data was expressed as the number of cases (as a percentage of the total).

P-value <0.05 was considered statistically significant.

*Significantly different from the naturally conceived group.

**Significantly different from the naturally conceived group and natural cycle group.

***Significant differences were found between any two groups.

### Placental disorders in natural conceived group and in the FET groups with different endometrium preparation regimens

The prevalence of abnormal placental thickness and intraplacental hematoma (such as placental lakes, placental hemangioma and placental abruption) was similar among the three groups. After adjusting for maternal age and parity, the prevalence of abnormal placental morphology, including sail-shaped placenta, multilobed placenta, racket placenta and accessory placenta, the incidence of abnormal placental position, including placenta previa and low-lying placenta, the incidence of abnormal placental attachment including placental adhesion and placenta increta, were similar between mNC-FET cycles and naturally conceived group. However, programmed-FET group was associated with a higher occurrence of abnormal placental morphology (aOR 5.27; 95%CI 2.00-16.44), abnormal placental position (aOR 3.84; 95%CI 1.82-5.82) and abnormal placental attachment (aOR 8.67; 95%CI 5.24-14.98) compared to natural conceived group. Overall, no significant difference was observed in the risk of placental disorders between mNC-FET cycles and natural conceived pregnancies (aOR 1.16; 95%CI 1.31-7.01), while programmed-FET cycles were associated with a higher occurrence of placental disorders (aOR 5.36; 95%CI 3.63-8.05) (**Table 3**).

**Table 3:** Unadjusted and adjusted odd ratios of placental developmental abnormalities in different cycles of frozen embryo transfer.

	Naturally conceived	Frozen embryo transfers							
	Reference group	Modified natural cycle				Programmed cycle			
Variables	(n=1334)	(n=217)				(n=635)			
		unadjusted		adjusted		unadjusted		adjusted	
		OR (95%CI)	*P*-value	OR (95%CI)	*P*-value	OR (95%CI)	*P*-value	OR (95%CI)	*P*-value
Abnormal placental morphology		14.39 (4.39-47.14)	<0.001	4.35 (4.14-6.84)	0.148	8.60 (2.86-25.81)	<0.001	5.27 (2.00-16.44)	0.002
Abnormal placental thickness		/	0.140	/	0.986	/	/	/	/
Abnormal placental position		3.39 (1.34-8.59)	0.016	3.83 (3.03-7.21)	0.143	3.48 (1.73-6.99)	<0.001	3.84 (1.82-8.52)	<0.001
Abnormal placental attachment		2.84 (1.28-6.33)	0.016	2.53 (2.08-4.73)	0.318	8.80 (5.32-14.55)	<0.001	8.67 (5.24-14.98)	<0.001
Intraplacental hematoma		1.0 (0.99-1.00)	1.000	/	0.973	1.26 (0.30-5.30)	1.000	0.51 (0.07-2.44)	0.427
Abnormal placental development		4.19 (2.51-6.99)	<0.001	1.16 (1.31-7.01)	0.879	6.80 (4.71-9.83)	<0.001	5.36 (3.63-8.05)	<0.001

Adjusted for: maternal age, parity.

We then focused on the dysfunction of placentation in programmed cycles (**Table 4**). Using the mNC-FET group as a reference and adjusting for confounders such as maternal age, parity, endometrial thickness, and number of embryos transferred, we found that abnormal placentation in programmed cycles mainly manifested as abnormal placental attachment, including placental adhesion and placenta increta (aOR 2.50, 95%CI 1.36-4.90). Additionally, this dysfunction of placentation in programmed cycles was independently associated with the type of infertility, the total dose of Femostone, and endometrial thickness.

**Table 4:** Unadjusted and adjusted odd ratios of significant placental pathology results.

Programmed versus modified natural cycle of endometrium preparation				
	unadjusted		adjusted	
Variables	OR (95%CI)	*P-value*	OR (95%CI)	*P-value*
Abnormal placental morphology	0.60 (0.26-1.37)	0.220	0.54 (0.22-1.34)	0.165
Abnormal placental thickness	1.00 (0.99-1.00)	0.225	NA	0.985
Abnormal placental position	1.01 (0.42-2.41)	0.983	0.65 (0.29-1.51)	0.294
Abnormal placental attachment	3.10 (1.52-6.29)	0.001	2.50 (1.36-4.90)	0.005
Intraplacental hematoma	1.00 (1.00-1.01)	0.575	NA	0.987
Abnormal placental development	1.63 (1.03-2.57)	0.036	1.37 (0.86-2.22)	0.192

Adjusted for: maternal age, parity, EMT (endometrial thickness), number of embryos transferred.

### Factors associated with abnormal placental phenotypes in programmed cycles

Since the incidence of placental disorders was higher in the programmed-FET group, we further analyzed the related factors (**Table 5**). Using univariate logistic regression analysis, we found that secondary infertility, older age, the addition of Femostone, the total dose of Femostone, and the thinner endometrium were correlated with the abnormal placental development. After analyzing these factors by multivariate logistic regression, the secondary infertility, the total dose of Femostone and thinner endometrium were still related to the prevalence of placental disorders.

Table 5Logistic regression analysis of factors associated with abnormal placental development in hormone replacement cycles.

**Table:** 

Univariate logistic regression analysis			
Variables	OR	95%CI	*P*
Primary infertility	0.43	0.28-0.67	<.001*
Age(years)	1.08	1.02-1.14	.004*
BMI (kg/m^2^)	0.97	0.91-1.04	0.473
AMH (ng/mL)	0.95	0.90-1.00	0.086
Progynova treatment	0.73	0.42-1.32	0.282
Total dose of Progynova(mg)	1	0.99-1.00	0.318
Addition of Femoston treatment	2.42	1.56-3.77	<.001*
Total dose of Femoston(mg)	1.01	1.01-1.02	<.001*
EMT (mm)	0.79	0.69-0.91	<.001*
Previous cesarean section	0.49	0.20-1.02	0.08

BMI, body mass index.

AMH, anti-Müllerian hormone.

EMT, endometrial thickness.

**Table:** 

Multivariate logistic regression			
Variables	adj.OR	95%CI	adj.*P*
Primary infertility	0.53	0.33-0.85	.008*
Age(years)	1.05	0.99-1.11	0.102
Total dose of Femoston(mg)	1.01	1.00-1.02	.014*
EMT (mm)	0.86	0.75-0.99	.038*

EMT, endometrial thickness.

*P-value <0.05 was considered statistically significant.

**Table:** 

		abnormal placental development	without abnormal placental development	*P*
Additon of Femoston treatment	n=139	50	89	<0.001
No addition of Femoston treatment	n=418	46	372	
EMT<8 mm	n=101	26	75	0.001
EMT≥8 mm	n=534	70	464	

EMT, endometrial thickness.

*P-value <0.05 was considered statistically significant.

### Association of different placental changes with the perinatal outcomes in programmed-FET cycles

In order to study the abnormal placental development on the incidence of maternal and neonatal complications, we divided the programmed FET group based on whether placental disorders had occurred (**Table 6**). Data revealed that, placental disorders in the programmed-FET group were associated with higher rate of preeclampsia, postpartum hemorrhage and Cesarean section, but were not related to the occurrence of gestational diabetes, premature birth, low birth weight or high birth weight.

**Table 6:** Association of abnormal placental development with the occurrence of perinatal complications in programmed cycles.

	abnormal placental development	without abnormal placental development	*χ^2^*	*P*
Variables	n=96	n=539		
Gestational diabetes	24(25.0)	156(28.9)	0.624	0.430
Preeclampsia	11(11.5)	28(5.2)	5.546	0.019
Premature birth	11(11.5)	36(6.7)	2.716	0.099
Cesarean section	83(86.5)	366(67.9)	13.545	<0.001
Postpartum hemorrhage	34(35.4)	61(11.3)	37.199	<0.001
Low birth weight	6(6.3)	19(3.5)	1.600	0.206
High birth weight	7(7.3)	56(10.4)	0.875	0.350

Categorical data was expressed as the number of cases (as a percentage of the total).

P-value <0.05 was considered statistically significant.

## Discussion

Our retrospective cohort study aimed to investigate the effect of different endometrial preparation protocols of FET on the placental development pattern in singleton live births. The placenta develops from the outer trophoblastic layer following the differentiation of the embryo and is more susceptible to epigenetic regulatory changes caused by environmental interventions and influences during assisted reproductive technology. Placenta not only regulates the development of the fetus, but also dysplasia of placenta will lead to poor maternal and perinatal outcomes as well as long-term health risks later in life, including neurodevelopmental disorders, tumors, and adult metabolic syndrome (10, 11). There is sufficient evidence that ART may be related to changes in placental morphology and structure, growth dynamics, imprinted and non imprinted genes, and other aspects that regulate placental formation (12, 13). Several studies have shown that the incidence of placenta previa in ART is significantly higher than in natural pregnancy (14). In addition, the placental weight of ART pregnancies is significantly larger, and the ratio of placental weight to birth weight is also higher (15). Some observations indicate that increased placental thickness during pregnancy obtained through ART leads to a higher incidence of hematoma (9). But few studies focus the different FET protocols on placentation. As a result, it is very meaningful to investigate the association between different intrauterous environment created by different endometrial preparation protocols on the placental changes and intrauterine fetal development.

When maternal age and parity did not take into consideration, the odds ratios of abnormal placental morphology, abnormal placental position and abnormal placental attachment were considerably higher from mNC-FET and programmed-FET groups compared to naturally conceived ones. However, after adjustment of maternal age and parity, the difference of abnormal placental disorder rate was more obvious only in the programmed-FET group compared to the spontaneously conceived pregnancies. Therefore, we speculated that part of the defective placental formation may be related to advanced maternal age.

Then we focus on the dysfunction of placentation in programmed cycles. Using mNC-FET group as reference and adjusting the cofounders like maternal age, parity, endometrium thickness, number of embryo transferred, we found that the abnormal placentation of programmed cycles mainly lied in abnormal placental attachment including placental adhesion and placental increta (aOR 2.50, 95%CI 1.36-4.90). Additionally, this dysfunction of placentation of programmed-FET cycles was independently associated with the infertility type, the total dose of Femostone and the endometrium thickness.

Infertility is often multifactorial, especially for primary infertility, with both male and female factors and a combination of genetic causes, environmental impacts and underlying disruption of hormonal and endocrine homeostasis. The etiology of primary infertility is different from secondary infertility, with a higher rate of unexplained infertility, ovulatory dysfunction, male factor and least common etiologies of tubal factor (16). Therefore, we speculate that the complicated causes behind primary infertility may be the reason related to placental development disorders. Secondly, in our study, we found that addition of Fenmotone was associated with an increased incidence of placental abnormalities. In our center, when the endometrium cannot meet 7mm using routine dosage of Progynova, we will add Femoston, which in turn resulted a higher cumulative estrogen dose and duration. This has been attributed to reduced endometrial thickness (17), but high-dose estrogen alone may also affect obstetric outcomes and placental findings. Recently published data also showed a similar phenomenon that the higher estrogen dose administered resulted in a higher rate of bilobated placentas, accessory lobes, accelerated villous maturation (18). Exposure to high estradiol concentrations may change the gene expression involved in endometrial remodeling (19). *In vivo* studies have shown that the placental junction region undergoes excessive growth and the ratio of fetus to placenta is low during superovulation, which may be related to decreased oxygenation and consequent placental dysfunction (20). However, research addressing this correlation in programmed cycles is still rare.

Abnormal placentation in the programmed-FET group is a cause of increased risk of preeclampsia, cesarean section, and postpartum hemorrhage. The link between programmed cycles and hypertensive disorders has gained a lot of attention during recent years (21). Programmed cycles lack corpus luteum, which produces several vasoactive molecules like relaxin, prorenin and other unknown molecules that contribute to the global changes that occur in early pregnancy and serve to reduce the risk of hypertensive disorders later in pregnancy (22, 23). At the same time, the prematurely elevated level of estradiol in programmed cycles may suppress the trophoblastic invasion of spiral arteries which may attribute to the increased risk of hypertensive disorders (24). Abnormal placentation, especially the increased rate of placental attachment, may be the result of a combination of the above factors, and is the origin of the complications of the obstetrical complications of postpartum hemorrhage and higher rate of cesarean section.

### Strengths and limitations

The study’s strengths lie in its novel approach to assess the placental morphology and structure to explore the effectiveness of different endometrial preparation protocol of FET cycles. The consistency of our study can also be assured as it was conducted in a single center, and placental analyses were carried out by the same pathologist blinded to patient background data. Additionally, multiple pregnancy was excluded, because multiple pregnancy only was reported as a risk factor for maternal and neonatal complications.

One limitation of our study is that it is a retrospective study, which may introduce biases and confounding factors. Although we adjusted for potential confounders, such as maternal age, parity, endometrium thickness, and number of embryos transferred, there may still be residual confounding factors that we did not measure or adjust for. In addition, the sample size of our study is relatively small, which may limit the generalizability of our findings. Further studies with larger sample sizes and prospective designs are needed to confirm our results. Moreover, according to the “developmental origins of health and disease” (DOHaD) hypothesis and the Barker hypothesis, placental dysplasia leads to poor perinatal outcomes as well as long-term health risks later in life (25, 26). It is necessary to perform a more rigorous assessment of placental function and also a follow-up on ART offspring after childbirth.

## Conclusion

In conclusion, our study suggests that programmed cycles of FET are associated with an increased risk of abnormal placental attachment, which may lead to obstetric complications such as preeclampsia, postpartum hemorrhage, and a higher rate of cesarean section. The high total dose of estrogen may also contribute to the abnormal placental development pattern in programmed cycles. Clinicians should be aware of these risks when counseling patients about their options for endometrial preparation for FET cycles. Further studies are needed to explore the underlying mechanisms and potential interventions to reduce the risk of abnormal placentation in programmed cycles.

## Data availability statement

The raw data supporting the conclusions of this article will be made available by the authors, without undue reservation.

## Ethics statement

The studies involving human participants were reviewed and approved by Reproductive Medicine Ethics Committee of Suzhou Municipal Hospital. The patients/participants provided their written informed consent to participate in this study.

## Author contributions

HL and QM supervised the entire study, including the procedures, design interpretation of the study data, and revisions of the article. FW: collected the data, drafted the manuscript and reviewed the manuscript. QW: collected the data, data analysis and drafted the article. YS: assessed the placenta. JD: collected the data. All authors contributed to the article and approved the submitted version.
